# Weekly versus triweekly cisplatin treatment in patients with locally advanced nasopharyngeal cancer during concurrent chemoradiotherapy

**DOI:** 10.1186/s40001-023-01297-y

**Published:** 2023-10-05

**Authors:** Xin Li, Lei Li, Ruimei Sun, Jingyan Gao, Zhengfei Li, Yongyuan Xue, Lixiu Zhu, Tianrui Xu, Chuanzheng Sun, Yan Xi, Wei Xiong

**Affiliations:** 1 Department of Radiotherapy, Yunnan Cancer Hospital, The Third Affiliated Hospital of Kunming Medical University, 519 Kunzhou Road, Kunming, Yunnan China; 2 Department of Head and Neck Surgery Section II, Yunnan Cancer Hospital, The Third Affiliated Hospital of Kunming Medical University, 519 Kunzhou Road, Kunming, Yunnan China

**Keywords:** Nasopharyngeal cancer, Concurrent chemoradiotherapy, Cisplatin, Meta-analysis

## Abstract

**Background:**

For patients with locally advanced nasopharyngeal cancer (LA-NPC), concurrent chemoradiotherapy (CCRT) is the standardized treatment. However, whether a weekly or triweekly cisplatin regimen should be used during CCRT is controversial. Therefore, we conducted this meta-analysis to explore differences in the effects and toxicities of the two regimens.

**Methods:**

We searched PubMed, Embase, and the Cochrane Library (until June 10, 2022). We evaluated overall survival (OS), distant metastasis-free survival (DMFS), locoregional recurrence–free survival (LRFS), disease-free survival (DFS) and grade ≥ 3 adverse events. The effect indices were hazard ratios (HRs) and odds ratios (ORs), and Review Manager software 5.4 (RevMan 5.4) was used for computations.

**Results:**

We identified 7 studies in our analysis. There was no significant difference in OS (HR = 1.00, 95% CI 0.73–1.38, P = 0.99), DMFS (HR = 0.84, 95% CI 0.58–1.22, P = 0.36), LRFS (HR = 0.91, 95% CI 0.63–1.32, P = 0.62) or DFS (HR = 0.93, 95% CI 0.56–1.56; P = 0.78) between the weekly and triweekly cisplatin regimens. We found that the weekly cisplatin regimen was more likely to cause grade ≥ 3 hematological toxicity events than the triweekly cisplatin regimen. In addition, subgroup analyses revealed that patients undergoing CCRT and CCRT plus adjuvant chemotherapy (AC) had similar OS or DFS.

**Conclusion:**

Weekly and triweekly cisplatin regimens had similar efficacy for LA-NPC. The triweekly regimen may replace the weekly regimen for LA-NPC because of lower toxicity. Larger data accumulation and more multicenter clinical trials may be needed to verify these results.

## Introduction

Nasopharyngeal cancer (NPC) is the most common primary neoplasm of the nasopharynx and is mainly found in Asia, especially in southern China. There are approximately 13,000 new cases of the disease diagnosed worldwide each year [1]. In addition, many patients are already in a locally advanced state at the time the disease is initially diagnosed. Locally advanced nasopharyngeal cancer (LA-NPC), to some extent, has a greater risk of locoregional relapse and distant metastases [2, 3]. Because it is sensitive to radiotherapy and chemotherapy, CCRT is the cornerstone of systemic treatment for patients with NPC. Good local control can be achieved in patients with LA-NPC who receive this therapy [4, 5]. Cisplatin-based therapies administered either once per week or once every three weeks are standard strategies for CCRT [6].

However, the two cisplatin regimens are more superior than other regimens. To date, the optimal schedule for cisplatin during CCRT is still controversial. Hence, the purpose of the meta-analysis was to compare survival outcomes and toxicities of the two different cisplatinum regimens for locally advanced NPC patients.

## Methods

### Literature search

We thoroughly searched PubMed, Embase, and the Cochrane library (each from inception to June 10, 2022) for literature studies. There was no language restrictions to identify studies. The search terms were constructed as described below: ‘nasopharyngeal neoplasm/carcinoma/cancer/tumor’, ‘nasopharynx neoplasms/carcinoma/cancer/tumor’, ‘concurrent chemoradiotherapy’, ‘chemoradiotherapy’, ‘cisplatinum’, and ‘cisplatin’. The above search terms were combined by using “AND” and “OR”. Qualified articles from the three medical databanks were searched independently by two team members. If there was any dispute, it was settled in a group discussion**.**

### Inclusion criteria

The included studies were required to satisfy the principles of PICOS (Population, Intervention, Comparison, Outcomes and Study design). The details are as follows: (1) P: patients with a pathological diagnosis of nasopharyngeal cancer; (2) I: patients received only cisplatin chemotherapy during CCRT. The experimental group received a triweekly cisplatin treatment, and the control group received a weekly cisplatin treatment; (3) C: Survival outcomes and toxicities were compared between the weekly and triweekly cisplatin regimens; (4) O: Studies with at least one reported outcome as follows: OS, DMFS, LRFS, DFS, and grade ≥ 3 toxicity (including hematological toxicity and nonhematological toxicity); (5) S: The study design consisted of randomized control trials (RCTs) and non-RCTs.

### Exclusion criteria

This meta-analysis had five exclusion criteria: (1) patients with distant metastases or severe coexisting illness; (2) Prior radiotherapy, chemotherapy, or clear primary neoplasms or lymph node surgical history; (3) lactation or pregnancy; (4) a significant difference in baseline data or no valuable information in the study; and (5) single arm studies, reviews, case reports, letters, comments or other unsuitable study types.

### Data extraction

Qualified articles from the abovementioned databanks were searched by two reviewers to determine whether they met the inclusion and exclusion criteria. Two team members read the selected articles to be included in this meta-analysis, and they were responsible for extracting relevant information according to the items in a standardized manner. Items were inventoried as follows: (1) baseline characteristics, including the first author’s name, country, published year, research period, median follow-up time, number of cases, study type, Eastern Cooperative Oncology Group Performance Status Scale (ECOG/PS) or Karnofsky's index of performance status (KPS), neoplasms clinical stage, intervention, comparisons, and patients’ ages and sexes; and (2) outcomes, including OS, DMFS, LRFS, DFS and grade ≥ 3 adverse events. We excluded articles with missing data. Disagreements were resolved in a panel discussion.

### Risk of bias and quality evaluation

Two researchers evaluated the risk of bias, and a third team member resolved differentials. The risk of bias in RCTs was evaluated by employing the Cochrane risk of bias tool, and the risk of bias in non-RCTs was evaluated by employing the Newcastle‒Ottawa Scale (NOS). The Cochrane risk of bias assessment tool has seven criteria, which are as follows: selection bias (including random sequence generation and allocation concealment), performance bias, detection bias, reporting bias, attrition bias and other bias. Three different levels (including high, low, or unclear risk bias) were used to evaluate each clause [7]. The NOS scale has the following three criteria: selection of experimental and control groups, comparability of experimental and control groups, and outcomes of research [8]. We gave a ‘star’ when we recognized the clause as ‘high ‘quality’. With the exception of the 'comparability' clause, which was allowed a maximum of two stars, the remaining clauses were allowed a maximum of one star. Study quality was classified as high level (7 ≤ stars ≤ 9), middle level (4 ≤ stars ≤ 6), and low level (1 ≤ stars ≤ 3).

### Statistical analysis

We used Review Manager software 5.4 (RevMan 5.4) to conduct this meta-analysis. We chose HR as the effect index, and the inverse-variance (IV) method was used to pool survival statistics [9]. Engauge Digitizer software was employed to extract HR from the survival curve when it could not be extracted immediately from qualified articles. Dichotomous variables were calculated by the odds ratio (OR), and the Mantel‒Haenszel (MH) method was used to evaluate the OR. We used χ^2^ and the I^2^ test statistic to examine heterogeneity. If the P value of the χ^2^ test was > 0.05 and I^2^ < 50%, the fixed-effect model was adopted for data with nonsignificant heterogeneity. Conversely, the random-effect model was employed due to significant heterogeneity. Moreover, to demonstrate the effect of AC, a subgroup analysis was conducted based on concurrent chemoradiotherapy.

The meta-analysis protocol was prospectively registered at PROSPERO (CRD42022341140).

## Results

### Study selection

**Fig. 1:**
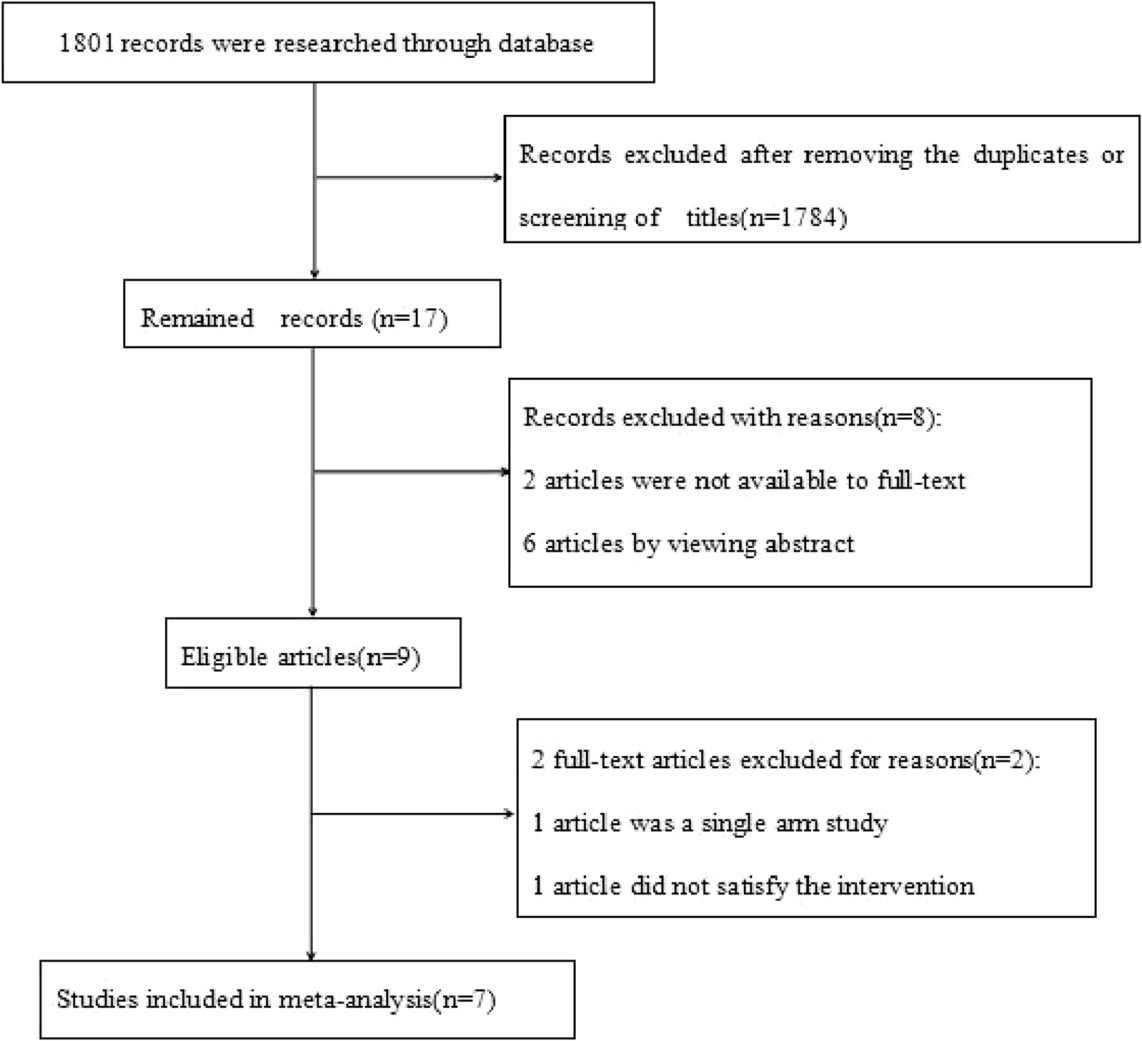
Flow chart of the study selection process

After we completed the relevant search, a total of 1801 articles were retrieved and records were excluded after removing duplicates or screening titles (n = 1784).Finally, 17 eligible articles remained. Ten articles were eliminated, and only 7 articles were included in this meta-analysis. The exclusion reasons were as follows: 2 articles were not available in full text, 6 articles were excluded after reviewing the abstract, 1 article was a single arm study, and 1 article did not satisfy the intervention. The entire process of study selection is displayed in Fig. 1**.**

### Features of the included studies

**Fig. 2:**
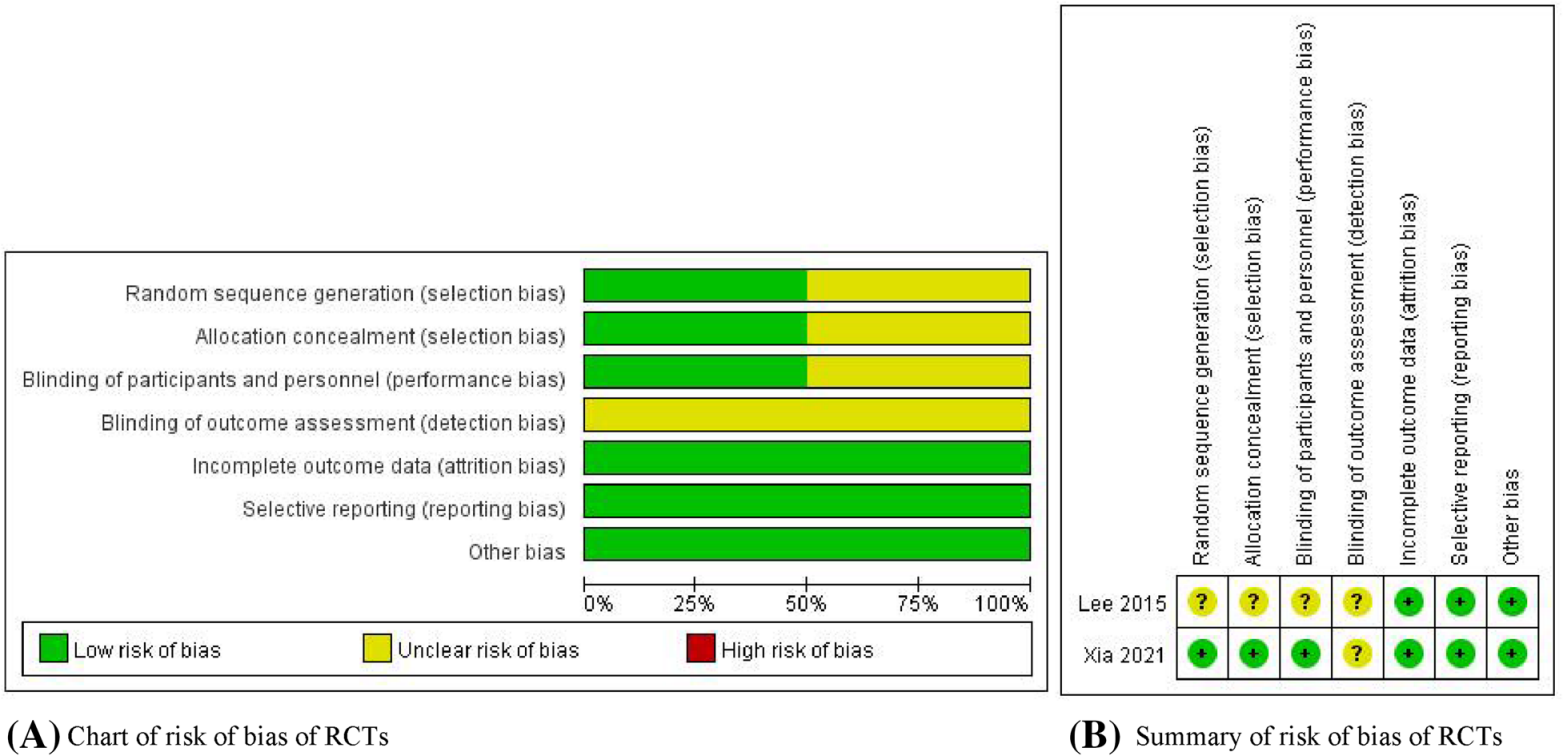
Risk of bias: retrospective authors’ judgments about each clause of risk of bias for RCTs

Ultimately, 7 studies with a total of 2151 patients were included in this meta-analysis. Among the 7 eligible articles, two [1, 10] were randomized controlled trials and five [6, 11–14] were retrospective studies. We used the Cochrane risk of bias tool to evaluate the quality of the two randomized controlled trials. Details are displayed in Figs. 2. We used the NOS scale to estimate the quality of the 5 retrospective studies, which were recognized as high level because all of them had 7 stars. Moreover, the basic information of the qualified studies contained in this meta-analysis is stated in Tables 1 and 2**.**

**Table 1:** Basic information of the included studies

Author (years)	Country	Research period	Study type	Meidian follow-up(months)	Sample size			Median age (W1,W3, y)	Sex(W1:M/F; W3:M/F)	Clinical stage	ECOG or KPS	Assessment of toxicity
					T	W1	W3					
Zhu 2018	China	2010–2013	Re	W1:51 W3:50	859	225	634	W1:45.2 W3:45	W1:170/55 W3:475/159	III–IVb (UICC 7th)	70–100	CTCAE 4.0
Xia 2021	China	2011–2016	RCT	58.3	510	250	260	W1:43 W3:44.5	W1:180/70 W3:177/83	III–IVb (AJCC 7th)	70–100	CTCAE 4.0
Wang 2019	China	2010–2013	Re	60.2	322	93	229	NA	W1:83/10 W3:217/12	I–Iva (AJCC 8th)	NA	CTCAE 4.0
Meng 2018	China	2008–2011	Re	69	180	90	90	W1:46 W3:41	W1:57/33 W3:57/33	III–IVb (AJCC 7th)	80–100	CTCAE 3.0
Lee 2015	Korea	2009–2013	RCT	30	109	53	56	W1:53.6 W3:52.7	W1:39/14 W3:47/9	II–IVb (AJCC 5th)	0–2	NA
Jagdis 2014	Canada	2000–2009	Re	W1:36 W3:72	73	45	28	W1:51 W3:49.5	W1:35/10 W3:15/13	II–IVb (UICC 7th)	0–3	CTCAE 3.0
Gundog2019	Turkey	2010–2018	Re	41.5	98	61	37	NA	W1:45/16 W3:25/12	II–Iva (AJCC 8th)	70–100	CTCAE 3.0

Re. :retrospetive study; RCT: randomized controlled trial; W1:weekly; W3:triweekly; T:total; y:years; M:male; F:female; AJCC: American Joint Committee on Cancer; UICC: The Union for International Cancer; ECOG: Eastern Cooperative Oncology Group; KPS: Karnofsky Performance Status; CTCAE: Common Terminology Criteria for Adverse Events; NA: Not available

**Table 2:** Treatment characteristics of the included studies

Author(years)	Concurrent chemoradiotherapy weekly regimen triweekly regimen		Median cumulative cisplatin dose weekly regimen triweekly regimen		Radiotherapy	Therapeutic schedule	Outcomes
Zhu (2018)	Cisplatin 40 mg/m^2^ based on oncologists’ opinions	Cisplatin 100 mg/m^2^d1,d22,d43	229.20 mg/m^2^	228.00 mg/m^2^	IMRT; dose:NA	CCRT alone	DFS,DMFS,LRRFS,OS
Xia (2021)	Cisplatin 40 mg/m^2^ for six cycles	Cisplatin 100 mg/m^2^ for two cycles	220.00 mg/m^2^	200.00 mg/m^2^	IMRT; ose:NA	CCRT alone	FFS,OS,DMFS,LRFS,ORR
Wang (2019)	Cisplatin 30–40 mg/m^2^ d1,d8,d15,d22,d29,d36, d43	Cisplatin 80–100 mg/m^2^ d1,d22,d43	190. 54 mg/m^2^	202.97 mg/m^2^	IMRT; dose:66–72 Gy	CCRT alone	OS,DFS,LRFS,DMFS
Meng (2018)	Cisplatin 30–40 mg/m^2^ weekly	Cisplatin 80 mg/m^2^ every 3 weeks	171.00 mg/m^2^	168.20 mg/m^2^	IMRT; dose:66–72 Gy	CCRT alone	OS,DFS,LRRFS, DMFS
Lee (2015)	Cisplatin 40 mg/m^2^ d1,d8,d15,d22,d29,d36, d43	Cisplatin 100 mg/m^2^ d1,d22,d43	248.90 mg/m^2^	256.60 mg/m^2^	3D-CRT or IMRT; dose:at least 66 Gy	CCRT + AC	PFS,OS,ORR,QOL, toxicity
Jagdis (2014)	Cisplatin 40 mg/m^2^ weekly for 7 week	Cisplatin 100 mg/m^2^ d1,d22,d43	230.00 mg/m^2^	249.00 mg/m^2^	3D-CRT or IMRT; dose:at least 66 Gy	CCRT + AC	OS,DFS
Gundog (2019)	Cisplatin 50 mg/m^2^ weekly	Cisplatin 100 mg/m^2^ every 3 weeks	NA	NA	2/3D-CRT or IMRT; dose:70 Gy	CCRT alone	OS,LRFS,DMFS, ORR

NA:not available; IMRT:intensity modulated radiotherapy; 3D-CRT:3-Dimensional conformalradiation therapy; 2D-CRT:2-Dimensional conformalradiation therapy; CCRT: concurrent chemoradiotherapy; AC:adjuvant chemotherapy; OS:overall survival; DFS:disease-free survival; DMFS:distant metastasis-free survival; LRRFS:loco-regional relapse-free survival; FFS:failure-free survival; LRFS: locoregional recurrence–free survival; ORR:overall response rate; PFS:progression-free survival; QOL: the European Organization for Research and Treatment of Cancer QOL questionnaire modules QLQ-C30 and QLQ-H&N35

### Primary endpoint

#### Overall survival (OS)

**Fig. 3:**
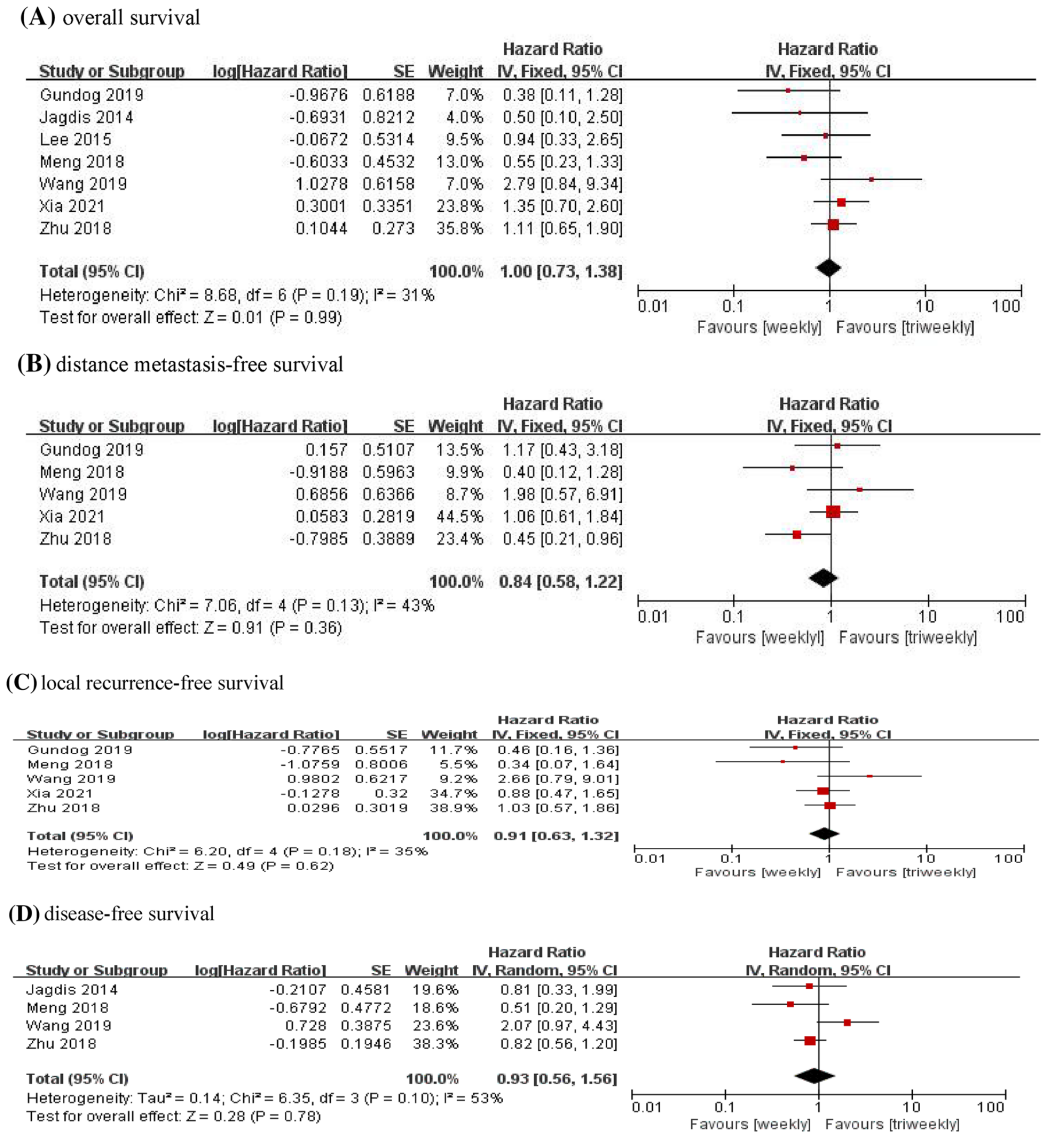
Forest chart for the survival outcomes of weekly cisplatin regimen comparing with triweekly cisplatin regimen

In all the studies [1, 6, 10–14] in our meta-analysis, OS data were reported and 817 patients received the weekly cisplatin regimen, whereas 1334 patients received the triweekly cisplatin regimen. Specifically, the OS data obtained by Meng [6] and Wang [12] were extracted from the results of the multivariate analyses, whereas the OS data obtained by Gundog [13] were extracted from the results of the univariate analyses. OS was very similar in patients receiving weekly and triweekly cisplatin regimens (pooled HR = 1.00, 95% CI 0.73–1.38, P = 0.99, Fig. 3A). A fixed-effect model was used since the heterogeneity test showed no significant difference (I^2^ = 31%, P = 0.19).

### Secondary endpoints

#### Distance metastasis-free survival (DMFS)

We could immediately extract the HRs of DMFS from 5 studies [1, 6, 11–13]. DMFS data obtained by Gundog [13] and Wang [12] were extracted from the results of the univariate analysis, and DMFS data obtained by Meng [6] were extracted from the results of the multivariate analysis. A fixed-effect model was used to compute pooled data due to no significant heterogeneity (I^2^ = 43%, P = 0.13). The results showed that the weekly and triweekly cisplatin groups had similar DMFS (pooled HR = 0.84, 95% CI 0.58–1.22, P = 0.36, Fig. 3B).

#### Local recurrence-free survival (LRFS)

LRFS data were reported in five articles [1, 6, 11–13], which contained a total of 1969 patients. It should be noted that LRFS data obtained by Gundog [13], Wang [12] and Meng [6] were from the results of the univariate and multivariate analyses, respectively. There was no significant difference between the two cisplatin regimens (pooled HR = 0.91, 95% CI 0.63–1.32, P = 0.62, Fig. 3C), with no heterogeneity (I^2^ = 35%, P = 0.18). Therefore, we employed a fixed-effect model.

#### Disease-free survival (DFS)

DFS data were reported in four studies [6, 11, 12, 14] with 1434 patients and used for our meta-analysis. Furthermore, the DFS data obtained by Meng [6] and Wang [12] were extracted from the results of the multivariate analyses. There was significant heterogeneity among these trials (I^2^ = 53%, P = 0.10); hence, a random-effect model was adopted to merge the data. The merged data revealed no statistically significant advantage for the weekly and triweekly cisplatin groups, with an HR of 0.93 (95% CI 0.56–1.56, P = 0.78, Fig. 3D).

#### Sensitivity and subgroup analysis

We performed a sensitivity analysis of this meta-analysis and found that all endpoints of the pooled results remained unchanged after removing each of the studies one by one. This suggests that the results of our meta-analysis are stable. In addition, we found two articles [10, 14] in which patients were treated with CCRT plus AC and other articles [1, 6, 11–13] in which patients were treated with only CCRT. Therefore, we designed a subgroup analysis. The results showed that OS and DFS were similar between the subgroups (details can be viewed in Table 3).

**Table 3:** The results of subgroup analysis

Outcomes	CCRT HR(95% CI)	P value	CCRT + AC HR(95% CI)	P value
OS	1.04 (0.74–1.47)	0.82	0.78 (0.32–1.86)	0.57
DFS	0.96 (0.48–1.92)	0.92	0.81 (0.33–1.99)	0.65

DFS: disease-free survival; OS: overall survival;CCRT: concurrent chemoradiotherapy; AC:adjuvant

chemotherapy;HR: hazard ratio;CI:confifidence interval

#### Treatment-related grade ≥ 3 adverse events

In 7 selected articles, researchers reported grade ≥ 3 adverse events, including hematologic toxic events (leukopenia, neutropenia, thrombocytopenia and anemia) and nonhematologic toxic events (nephrotoxicity/renal dysfunction, nausea/vomiting/constipation/diarrhea, skin reaction/dermatitis/rash, mucositis/stomatitis, xerostomia, and ototoxicity). Acute toxicity was evaluated according to the Common Terminology Criteria for Adverse Events (CTCAE). As shown in Table 4, the weekly cisplatin regimen was significantly associated with thrombocytopenia (pooled OR = 3.49, 95% CI 1.98–6.16, P < 0.0001), leukopenia (pooled OR = 1.50, 95% CI 1.16–1.93, P = 0.002) and neutropenia (pooled OR = 1.48, 95% CI 1.02–2.15, P = 0.04) compared to the triweekly cisplatin regimen. There was no statistically significant difference in the other adverse events between the two cisplatin groups.

**Table 4:** Odds ratios (ORs) of treatment-related grade ≥ 3 adverse events

Advese event (grade ≥ 3)	Trials (N)	Availability		Effect OR(95% CI)	P value	Heterogeneity		Analysis model
		Weekly (events/total)	Triweekly (events/total)			I^2^ value	P value	
Haematological								
Anaemia	5	19/710	17/1269	1.63 (0.84–3.15)	0.15	24%	0.26	Fixed effect
Thrombocytopenia	6	40/755	19/1297	3.49 (1.98–6.16)	< 0.0001	0%	0.78	Fixed effect
Neutropenia	4	64/572	69/978	1.48 (1.02–2.15)	0.04	0%	0.87	Fixed effect
Leukopenia	4	139/657	179/1213	1.50 (1.16–1.93)	0.002	0%	0.46	Fixed effect
Non-haematological								
Nephrotoxicity/Renal dysfunction	5	3/702	2/1241	1.37 (0.32–5.81)	0.67	29%	0.24	Fixed effect
Nausea/Vomiting/Constipation/Diarrhea	7	124/816	240/1334	0.83 (0.41–1.68)	0.61	76%	0.0003	Random effect
Skin reaction/Dermatitis/Rash	6	33/771	41/1306	1.00 (0.62–1.62)	1	0%	0.56	Fixed effect
Mucositis/Stomatitis	7	188/816	280/1334	0.87 (0.69–1.09)	0.23	46%	0.08	Fixed effect
Xerostomia	2	16/339	20/350	0.83 (0.42–1.62)	0.58	0%	0.56	Fixed effect
Ototoxicity	3	3/387	7/517	0.51 (0.14–1.87)	0.31	0%	0.86	Fixed effect

OR: odds ratio; CI: confidence interval

## Discussion

As described above, survival outcomes (including OS, DFS, DMFS, and LRFS) were similar between weekly cisplatin and triweekly cisplatin regimens during the CCRT period. However, it should be noted that the weekly cisplatin regimen had a higher incidence rate of grade ≥ 3 acute hematological toxic events, particularly in terms of thrombocytopenia and leukopenia. Subgroup analysis revealed no significant difference in OS or DFS between CCRT and CCRT plus AC. This suggests that patients with locally advanced nasopharyngeal cancer may not benefit from AC.

According to Chen’s research, CCRT could improve the overall survival (OS) and progression-free survival (PFS) of nasopharyngeal carcinoma patients compared with radiotherapy alone in the era of traditional 2D-RT [15]. The subsequent intergroup 0099 randomized trial confirmed that CCRT was better than radiotherapy alone for patients with locally advanced nasopharyngeal cancer (LA-NPC) [16]. Several meta-analyses have already shown that CCRT combined with or without AC could significantly improve OS [17–20]. Thus, CCRT has become the core therapy for patients with locally advanced nasopharyngeal cancer (LA-NPC). However, in the field of concurrent cisplatin dose delivery, either once a week or every 3 weeks, we found that the two regimens are popularly used in clinical practice but lack high-quality comparable evidence. Therefore, we performed this meta-analysis and discovered no statistical differences in survival outcomes between the two regimens, which was consistent with results from two other meta-analyses of cisplatin regimens in head and neck carcinoma [21, 22]. The possible reasons for the above results are as follows: First, for NPC, radiotherapy is the cornerstone, and radiotherapy alone may already achieve good local control. Second, during radiotherapy combined with cisplatin chemotherapy, whether the weekly or triweekly regimen both can improve radiotherapy sensitivity, eliminate micrometastases and prolong survival. Third, there is some evidence to suggest that the cumulative dose of cisplatin during CCRT is more meaningful than the administration schedule. Some studies have shown that good efficacy can be achieved if the cumulative dose of cisplatin is no less than 200 mg/m^2^ [23]. In our meta-analysis, except for the study by Meng [6] and Gundog [13], and the median cumulative dose of cisplatin in other studies [1, 10–12, 14] all reached or exceeded 200 mg/m^2^.

Although there were no significant differences in survival outcomes of the weekly and triweekly cisplatin regimens,through this meta-analysis, we observed differences in hematological adverse events in the two groups. Contrasting with the triweekly cisplatin regimen, the weekly cisplatin regimen obviously led to more thrombocytopenia and leukopenia. The reason for this result may be that there was a short interval between the weekly cisplatin regimen, and patients who had not recovered from previous chemotherapy and received concurrent radiotherapy at the same time were more prone to develop hematotoxicity. However, the report by Lee suggested similar toxicity between the two groups [10]. Furthermore, Rampino believed that more frequent administration of smaller dosages of cisplatin would cause less toxicity when preserving the therapeutic effect [24]. Nonetheless, we believe that our meta-analysis is more convincing because it included RCTs and retrospective studies with large sample sizes. Of course, an increasing level of exploration is warranted in the future.

Our subgroup analysis showed that AC was not associated with efficacy. CCRT was close to CCRT + AC in terms of OS and DFS for patients with locally advanced nasopharyngeal cancer. This finding is consistent with several published studies. Two clinical trials revealed that AC did not significantly improve OS or PFS [25, 26]. In addition, two meta-analyses showed that AC after CCRT did not improve survival [27, 28]. Regarding CCRT plus AC, patients have more serious toxicity, resulting in poor tolerance and compliance. Thus, only some of the patients can undergo AC in most cases, which may be the reason why AC cannot increase survival benefits [29, 30].

It is worth noting that in our included studies, whether using a weekly cisplatin regimen or triweekly cisplatin regimen, most patients received radiotherapy with intensity-modulated radio therapy (IMRT) technology, while only a few patients received radiotherapy with 2D-RT or 3D-RT technology. With the improvement of radiotherapy technology, IMRT technology has become mainstream for nasopharyngeal cancer patients by degrees. In two studies, it was suggested that IMRT improves local control for nasopharyngeal carcinoma compared to 2D-RT [31, 32]. Another study compared the 10-year survival outcomes of 2D-RT with IMRT, showing that IMRT improved OS and DFS in nasopharyngeal carcinoma patients [33]. However, we did not focus on radiotherapy techniques in our statistical analysis. The reason was that we thought that 2D-RT or 3D-RT may be able to achieve the optimal radiotherapy effect for patients with LA-NPC. Furthermore, the multivariate Cox analyses of two studies discovered that different radiotherapy techniques were not significantly related to survival outcomes [34, 35]. Therefore, more research is required to identify the best radiotherapy techniques in the future.

Moreover, the 2022 American Society of Clinical Oncology (ASCO) annual meeting has already reported that IMRT alone can achieve similar survival rates compared to CCRT for low-risk stage II nasopharyngeal carcinoma, and it can decrease toxicity and increase quality of life [36]. However, in our meta-analysis, some studies included stage II patients, but risk stratification was not performed, and all patients with stage II received CCRT treatment. It may be necessary for the future to further classify patients with stage II NPC and to use different treatments for different types to maximize the patient's benefit.

Nonetheless, there were some limitations in our meta-analysis. First, this meta-analysis had only two RCTs, and the others were retrospective studies. Second, most of the studies came from China, which has a high incidence of nasopharyngeal cancer, and it is not clear whether our results are applicable to other regions. Third, not all articles showed data on LRFS, DFS and DMFS. Fourth, the level of radiotherapy varies among centers. Finally, different studies had different median follow-up times.

## Conclusion

The weekly cisplatin regimen showed no difference in survival outcomes but more hematological toxicity in the treatment process than the triweekly cisplatin regimen. We speculate that the triweekly regimen has the potential to replace the weekly regimen for locally advanced nasopharyngeal cancer in the future, although larger data accumulation and more multicenter clinical trials may be needed to verify these results.

## Data Availability

The datasets used and/or analysed during the current study are available from the corresponding author on reasonable request.

## Abbreviations

LA-NPC: Locally advanced nasopharyngeal cancer
CCRT: Concurrent chemoradiotherapy
OS: Overall survival
DMFS: Distant metastasis-free survival
LRFS: Locoregional recurrence–free survival
DFS: Disease-free survival
HR: Hazard ratio
OR: Odds ratio
RevMan 5.4: Review Manager software 5.4
AC: Adjuvant chemotherapy
NPC: Nasopharyngeal cancer
PICOS: Population, Intervention, Comparison, Outcomes and Study design
RCT: Randomized control trial
ECOG/PS: Eastern Cooperative Oncology Group Performance Status Scale
KPS: Karnofsky's index of performance status
NOS: Newcastle‒Ottawa Scale
MH: Mantel‒Haenszel
ASCO: American Society of Clinical Oncology
Re.: Retrospetive study
AJCC: American Joint Committee on Cancer
UICC: The Union for International Cancer
CTCAE: Common Terminology Criteria for Adverse Events
NA: Not available
IMRT: Intensity modulated radiotherapy
3D-CRT: 3-Dimensional conformalradiation therapy
2D-CRT: 2-Dimensional conformalradiation therapy
LRRFS: Loco-regional relapse-free survival
FFS: Failure-free survival
ORR: Overall response rate
PFS: Progression-free survival
QOL: The European Organization for Research and Treatment of Cancer QOL questionnaire modules QLQ-C30 and QLQ-H&N35
CI: Confifidence interval
